# Effects of Maternal Grape Juice Intake on Unfolded Protein Response in the Mammary Glands of Offspring of High Fat Diet Fed Rat Dams

**DOI:** 10.3390/nu12082253

**Published:** 2020-07-28

**Authors:** Caroline Dani, Luciana Kneib Gonçalves, Isabel Teixeira Proença, Fabia de Oliveira Andrade, Leena Hilakivi-Clarke

**Affiliations:** 1 Master of BioScience and Rehabilitation, Methodist Center IPA, Porto Alegre, RS 90420-060, Brazil; caroline.dani@ipa.metodista.br (C.D.); l.kneib@yahoo.com.br (L.K.G.); isabel.proenca@ufrgs.br (I.T.P.); 2 Department of Oncology, Georgetown University, Washington, DC 20057, USA; fd265@georgetown.edu

**Keywords:** maternal diet, grape juice, high fat diet, prevention, offspring

## Abstract

Maternal high fat diet (HFD) and obesity during pregnancy increase female offspring′s mammary cancer risk in animal studies. We aimed to observe whether the consumption of grape juice during pregnancy can reverse this risk. During pregnancy and lactation, female Wistar rats were fed either a control or HFD and also received grape juice or tap water. At the age of 50 days, female offspring were euthanized, and mammary glands were collected to assess changes in biomarkers of increased mammary cancer risk. Maternal HFD increased the number of terminal end buds in offspring’s mammary glands and promoted cell proliferation (ki67). Maternal grape consumption blocked these effects. Apoptosis marker caspase 7, but not caspase 3, was reduced in the HFD offspring. HFD offspring also exhibited a reduction in the indicators of cell cycle regulation (p27, p21) and an ability to maintain DNA integrity (reduced p53). Maternal grape juice did not have any effect on these endpoints in the HFD offspring but reduced caspase 7 and p53 levels in the control offspring, perhaps reflecting reduced cellular stress. Maternal HFD increased oxidative stress marker GPx1 mRNA expression, and grape juice increased the levels of GPx2 in both the control and HFD offspring. HFD increased XBP1/Xbp1s, Atf4 and Atf6 mRNA expression and reduced ATF6 and CHOP protein levels. Maternal grape juice reversed the increase in XBP1/Xbp1s, Atf4 and Atf6 in the HFD offspring. PPARγ was downregulated in the HFD group, and grape juice reversed this effect. Grape juice also reduced the levels of HER2 and IRS, both in the control and HFD offspring. In conclusion, maternal grape juice supplementation reversed some of the biomarkers that are indicative of increased breast cancer risk in the HFD offspring.

## 1. Introduction

Breast cancer is the most common invasive cancer among women, comprising 30% of all female cancers in the United States [1]. However, the incidence of breast cancer varies in different regions across the world. In Asian countries, the cumulative risk of developing breast cancer at the age of 75 is 3.0%, whilst in the U.S., it is 10.0% [2]. The differences in risk are not caused by ethnicity: over two generations, descendants of Asians who migrated to the west have acquired the same breast cancer incidence as the population of the host country [3,4]. Environmental and lifestyle factors may thus have a major role in determining breast cancer risk.

Several studies indicate that maternal diet during pregnancy alters daughters′ breast cancer risk. In humans, high birth weight that often reflects high maternal fat intake and obesity [5] increases daughters′ breast cancer risk [6,7,8]. We have found that maternal intake of a high fat diet (HFD) composed of corn oil, which contains n-6 polyunsaturated fatty acids (PUFAs), or of cottonseed oil Crisco, which contains saturated fats and PUFAs, was associated with increased mammary cancer risk among the female offspring [9,10,11]. Similar increases in offspring′s mammary cancer risk as a result of maternal HFD have been seen in many different preclinical breast cancer models that include mice developing spontaneous mammary tumors, genetically modified mice and carcinogen-treated rats [12,13].

The exact mechanisms mediating the effects of maternal HFD in increasing daughters’ breast cancer risk remain to be identified. Maternal HFD and obesity may alter pregnancy hormonal or inflammatory environment [14,15], which then could epigenetically preprogram mammary gland development to include more targets for malignant transformation, i.e., mammary stem cells in terminal end buds (TEBs) [11,16,17]. In utero HFD exposure also alters gene signaling pathways that are linked to increased risk of malignant transformation [10,11]. Inflammation in the pregnant dam can cause oxidative damage and unfolded protein response (UPR) in the offspring [18]. Maternal obesity is found to induce a persistent activation of the UPR in the offspring′s hypothalamus, liver and adipose tissue [19,20], but not in the pancreas [21]. UPR is a cellular stress response that initially maintains normal physiological functions and protects the cells from irreversible damage. However, when UPR is chronically activated, it increases breast cancer risk [22,23]. It is not known if maternal HFD increases UPR in the offspring′s mammary glands.

We studied here whether maternal HFD might also affect UPR in the offspring′s mammary glands. UPR response is composed of three arms: (1) PKR-like EnR regulation kinase (PERK) which phosphorylates eukaryotic initiation factor 2α (EIF2α) and activates activating transcription factor 4 (ATF4) and DNA damage inducible transcript 3/CHOP, resulting in the inhibition of protein synthesis, induction of apoptosis and/or modification of antioxidant responses [23]; (2) ATF6 that regulates expression of UPR targets, and (3) inositol requiring enzyme 1α (IRE1α), which splices inactive X-box binding protein 1 (XBP1) to produce active spliced XBP1 [24]. XBP1 controls estrogen-induced estrogen receptor positive (ER+) cancer cell growth [25] and survival by modulating the nuclear factor kappa B (NFkB) pathway [26] and promotes antiestrogen resistance [27]. IRE1α and ATF6α activation causes induction of the chaperone BiP/GRP78/HSPA5 to increase protein-folding capacity and degradation of misfolded proteins [28].

Since maternal obesity not only increases daughters’ breast cancer risk [6,7,15] but has multiple other adverse effects on offspring, including increasing susceptibility to metabolic and cardiovascular diseases [29], several studies have investigated whether these adverse effects can be prevented by interventions during pregnancy. Among these interventions are giving pregnant dams natural compounds, such as resveratrol and germinated brown rice plus gamma-amino butyric acid (GABA) extract, taurine or adiponectin, or lifestyle interventions such as exercise [30]. Wu et al. [19] investigated whether maternal exposure to quercetin prevents an increase in UPR in offspring. Quercetin is present in many food products, such as grapes, berries, apples, cruciferous vegetables and red onions. The results of the study indicated that maternal quercetin supplementation prevented high birth weight and postnatal excessive body weight gain in the offspring of obese dams, improved their insulin sensitivity and lipid metabolism and reduced endoplasmic reticulum (EnR) stress and UPR as well as related inflammation in the liver and adipose tissue [19].

Among foods and beverages, purple grape juice is considered one of the richest sources of polyphenols, such as flavonoids, tannins and resveratrol [31]. In experimental models, grape juice has been shown to prevent platelet aggregation, low-density lipoprotein oxidation, oxidative damage to the DNA and brain and coronary artery diseases [32,33,34,35,36]. Specifically, exposure to grape juice reduced the oxidative damage in the brain and liver caused by adult consumption of HFD [33,37]. In a study by Buchner et al. [33], HFD-induced hepatocellular degeneration and steatosis was prevented by grape juice. Other proposed mechanisms for grape juice in preventing cancer are its ability to inhibit aromatization of testosterone to estrogens and to reduce angiogenesis [38].

The present study was designed to evaluate whether maternal intake of red grape juice by pregnant and nursing rat dams fed an HFD reverses biomarkers of increased mammary cancer risk among offspring. For this purpose, changes in TEBs, cell proliferation, apoptosis and markers of oxidative damage and activation of UPR were assessed in the offspring’s mammary glands.

## 2. Methods

### 2.1. Maternal Dietary and Fluid Exposures

Forty female and 20 male Wistar rats which were 90 days of age were obtained from the bioterium of the IPA Methodist University in Brazil. Rats were divided into two groups: those fed a high fat diet (HFD) (n = 20 females and 10 males) and those fed a control diet (20 females and 10 males). The HFD was acquired from Pragsoluções Biosciences (Jau, Sao Paulo, SP, Brazil), and its composition was as follows: casein as protein (20.0%), corn starch (14.95%), dextrinized starch (10.0%) and saccharose (10.0%) as carbohydrates, lard (31.0%) and soy bean oil (4.0%) as fat, microcrystalline cellulose (5.0%), L-cystine (0.30%), choline bitartrate (0.25%), butylated hydroxytoluene (BHT) (0.0010%), AIN93G mineral mix (3.50%) and AIN93G vitamin mix (1.00%). Control diet was acquired from NUVILAB CR1, and its composition was as follows: protein (25.0%), carbohydrate (58.0%), fat (5.0%). The vitamins in this diet were vitamin A (13,000 IU), D3 (2000 IU), E (34 mg), K3 (3 mg), B1 (5 mg), B2 (6 mg), B6 (7 mg), B12 (22 mcg), niacin (60 mg), folic acid (1 mg), biotin (0.05 mg) and choline (1900 mg).

Rats were housed in the ratio of 2 females per one male (2: 1) and mated. When the presence of spermatozoid was confirmed, the female rats consuming control or HFD were divided into two additional groups: those which had purple grape juice as their sole fluid source or those drinking tap water. Purple grape juice of Vitis labrusca L. variety Bordo was kindly provided by Adega Fante (Flores da Cunha, RS, Brazil). The juice was from the 2015 harvest, and all juice used in our study was from the same lot. In our previous analysis [39], it was found to contain phenolic compounds (17.87 ± 0.28 mg/dL), resveratrol (0.24 ± 0.01 mg/dL), catechin (1.17 ± 0.01 mg/dL), epicatechin (5.02 ± 0.06 mg/dL) and naringin (38.23 ± 0.48 mg/dL).

We have previously reported that maternal intake of the energy-dense HFD used in our study did not lead to an increase in pregnancy weight gain in Wistar rats [39]. We determined whether this was caused by reduced food intake by HFD offspring. Our results indicated that the HFD fed dams ate less (11.51 ± 0.63 g/day) than control diet fed dams (18.76 ± 0.54 g/day, *p* = 0.003). Grape juice intake further reduced food intake in the HFD dams (9.09 ± 1.54 g/day, *p* = 0.016 compared with no grape juice HFD dams) and control diet dams (13.42 ± 1.54 g/day, *p* = 0.023 compared with no grape juice control diet dams). Based on these intakes, the average daily intakes of carbohydrates, protein and lipids of pregnant and lactating rats consuming control or HFD and drinking either tap water or grape juice are shown in Table S1. Table S2 shows the average daily intake of phenolic compounds from grape juice in rats consuming control or HFD. Of all the bioactive compounds present in the grape juice, rats consumed the most rutin and chlorogenic acid.

After offspring were weaned, they were all fed the control diet and tap water. No significant differences in offspring′s postweaning weight gain or oxidative damage markers in the offspring′s liver were seen (Figure S1).

Prior to mating, throughout pregnancy and during lactation, rats had free access to their respective diet and were maintained in a 12-hr light–dark cycle, at a temperature of 22 ± 1°C. When offspring were weaned, they were all switched to the control diet and only received tap water to drink. The entire experiment was conducted with the approval of the Ethics Committee on Animal Use (ECAU) of the Methodist University Center—IPA (number 009/2014).

### 2.2. Offspring′s Mammary Gland Morphology

When offspring were 50 days of age, ten offspring per group were euthanized and mammary glands were collected. The right 4th abdominal mammary glands of the offspring of dams given control diet (CO), high fat diet (HFD), grape juice in control diet (GJ) and grape juice in high fat diet (HFDGJ) were obtained and prepared for a wholemount to assess the number of terminal end buds (TEBs) and epithelial tree elongation. Mammary tissue was placed on a glass slide and fixed overnight with a solution containing 6 parts 100% ethanol, 3 parts chloroform and 1 part glacial acetic acid. Then, the slides were washed in 70%, 50% and 30% ethanol for 15 min total, in running water for 5 min and incubated with carmine (Sigma-Aldrich) overnight. Tissues were washed again in running water and in 90%, 95% and 100% ethanol and put in xylene until there was no fat seen in the mammary tissue. TEBs were identified based on their size and localization and accessed in the periphery of the epithelial tree. Ductal elongation was measured as the distance between the nipple and the longest edge of epithelial outgrowth towards the end of the mammary fat pad.

### 2.3. Immunohistochemistry (IHC) for Ki67 in Mammary Glands

Another group of 50-day-old offspring was euthanized, and their mammary glands were collected to perform the assays described below. Antigen retrieval was achieved using Tris/EDTA, pH 9.0, for 20 min at 100 °C, followed by a 20-min cooling period. Mammary gland sections (*n* = 4–7/group) were incubated in Ki67 primary antibody (Novus Biologicals, Littleton, CO, USA, NB600-1252) at a dilution of 1:40 for 1 h. Subsequently, the sections were incubated in Horseradish Peroxidase (HRP)-rabbit secondary antibody (Dako, K4003) for 30 min, followed by diaminobenzidinetetrahydrochloride (DAB) and counterstaining with hematoxylin. The protein buffer was used as a negative control. Scoring was based on the percentage of positive staining of the overall tissue. The images were measured by Image J software.

### 2.4. Total Protein Extraction

For the extraction of proteins, 100 mg of mammary gland was homogenized with 200 mL of Radioimmunoprecipitation assay buffer (RIPA buffer) (150 mM NaCl, 50 mM Tris base pH 7.5, 1% Igepal CA-630, 0.5% Na deoxycholate), with protease inhibitor cocktail (Complete Mini, Roche, 1 tablet for 10 mL of RIPA buffer) and 1 mM sodium orthovanadate, 1 mM phenylmethylsulfonyl fluoride (PMSF), 10 mM glycerophosphate and 250 mM sodium pyrophosphate. The solution was kept on ice for 30 min and centrifuged for 10 min at maximum speed. The supernatant was transferred to a new tube and protein quantification was analyzed with the BCA Protein Assay (Thermo Scientific).

### 2.5. Western Blotting

Proteins were separated by electrophoresis (SDS-PAGE) in a 4–12% polyacrylamide gel (WG1402, Life Technologies, Carlsbad, CA, USA) and tris-glycine 1x buffer, using a vertical electrophoresis tank (XCell4 SureLock™ Midi-Cell, Life Technologies, Carlsbad, CA, USA). Proteins were then transferred from the gel to a nitrocellulose membrane with the iBlot2 system (IB23001, Life Technologies, Carlsbad, CA, USA). The membrane blocking was performed by non-fat milk, diluted in Tris Buffered Saline with Tween^®^ (TBS-T) (5%) for 1 h at room temperature, under stirring. After blocking, the membrane was incubated with the primary antibody, diluted in TBS-T (1:1000) overnight at 4 °C. The membrane was then incubated with a secondary antibody diluted in milk 5% (1:5000) for 1 h. For control, the membrane was incubated with anti-actin antibody (1:1000) overnight at 4 °C under stirring and then with goat secondary antibody (1:10000) for 1 h at 37 °C under stirring. Immunodetection was performed using the Chemiluminescent HRP Antibody Detection reagent (E2400, Denville Scientific Inc., Metuchen, NJ, USA) and the signal captured using film (eba45, Radiomat). All the antibodies used are listed in Table S3.

### 2.6. Evaluation of mRNA Expression

The mRNA expression of genes related to oxidative stress and UPR genes were evaluated by the real-time PCR technique in the left 4th mammary gland of the 50-day-old female offspring of rat dams exposed to the four different conditions (CO, HFD, GJ and HFDGJ) during gestation. These glands were obtained from the same rats used to assess mammary gland morphology.

We extracted total RNA using the RNeasy Lipid Tissue Mini Kit (Qiagen), according to the company protocol. The reverse transcription was performed according to the protocol of the High Capacity cDNA Reverse Transcription Kit (Life Technologies, Carlsbad, CA, USA). We performed the expression quantification of mRNA from the selected genes by real-time PCR, using the Sybr green amplification system (QuantiFast SYBR Green PCR Kit, Qiagen, Germantown, MD USA). PCR reactions were performed on Applied Biosystems 7900HT Fast Real-Time PCR System (Applied Biosystems, Foster City, CA, USA). The Ct (threshold) values obtained were used to determine the relative mRNA expression of each target gene relative to the *Gapdh* (endogenous control). We chose *Gapdh* after testing *Actin*, *Hprt*, *Gapdh* and *Tpb* to be used as an endogenous control, using GENORM. All the primers used were obtained from IDT (Integrated DNA Technologies, Coralville, Iowa, USA) and are listed in Table S4.

### 2.7. Statistical Analyzes

Data were first tested for normal distribution (Shapiro–Wilk test) and variance homogeneity (Levene). Differences between the groups were evaluated by two-way ANOVA, followed by Sidak′s test as a post hoc assessment tool. Results are expressed as a mean ± SEM, and the differences were considered statistically significant if *p* < 0.05. Sigma Stat (StatSoft) was used to analyze the data.

## 3. Results

### 3.1. Effects of Maternal Grape Juice Intake on Biomarkers of Increased Breast Cancer Risk in the Offspring′s Mammary Glands: TEB, Cell Proliferation and Apoptosis

**Figure 1:**
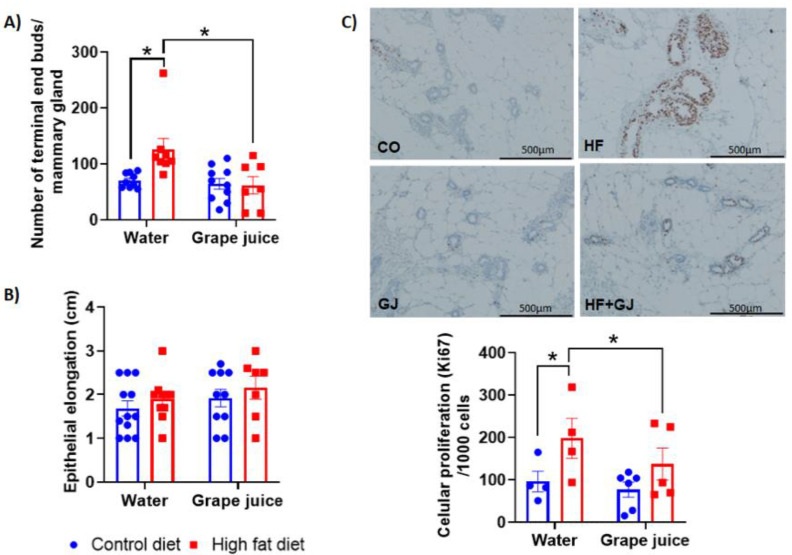
Mammary gland morphology in 7-week-old female rat offspring of dams fed control or high fat diet (HFD) during pregnancy and lactation. These dams either received tap water or grape juice. Quantitated data and Western blots are shown. (**A**) Number of terminal end buds (TEBs) per mammary gland. (**B**) Epithelial elongation (in cm) of mammary gland. Photomicrography (40×) of Ki67 staining and quantification of cell proliferation (**C**) in mammary glands of female offspring from the different groups. * Statistically significant difference (*p* ≤ 0.05) according to two-way ANOVA followed by Sidak′s test. The data are expressed as the mean ± SEM.

Mammary gland morphology: terminal end buds. Offspring of dams consuming HFD during pregnancy and lactation exhibited more TEBs than control offspring when assessed at 50 days of age (*p* = 0.03) (Figure 1A). Grape juice prevented this increase in the HFD offspring (*p* = 0.02) (Figure 1A). Ductal elongation was not significantly different between the control and HFD offspring, and maternal grape juice intake did not affect this endpoint (Figure 1B). Since an increase in TEBs at the age of 50 days in rats is associated with increased mammary cancer risk [40], our results suggest that HFD offspring were at increased mammary cancer risk and maternal grape juice intake prevented this increase.

Cell proliferation: Ki67. The effects of maternal HFD and grape juice intake on cell proliferation were studied in the mammary glands of 50-day-old offspring. Cell proliferation, assessed by determining the number of Ki67 positive cells by immunohistochemistry, was significantly increased in the mammary glands of HFD offspring (*p* = 0.019) (Figure 1C). Maternal grape juice significantly reduced the number of Ki67 cells in the HFD group (*p* = 0.033) but had no effect on Ki67 marker in the control offspring.

**Figure 2:**
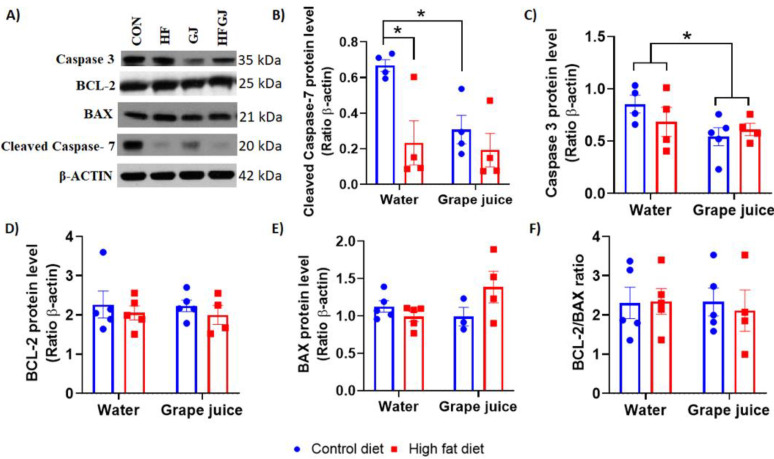
(**A**) Representative Western blots and quantitated data of (**B**) cleaved caspase-7, (**C**) cleaved caspase-3, (**D**) BCL-2, (**E**) BAX and (**F**) the BCL-2/BAX (**E**) protein ratio in mammary glands of 7-week-old female offspring of dams fed control or HFD and given tap water or grape juice. Caspase 7 levels were significantly lower in the HFD offspring when their dams had been drinking tap water. None of the other apoptosis markers were affected by maternal diet or grape juice. The data are expressed as the mean ± SEM. * Statistically significant difference (*p* ≤ 0.05) according to two-way ANOVA followed by Sidak′s test.

Apoptosis: caspases 3 and 7. Apoptosis was assessed by determining cleaved caspases 3 and 7. BCL2 family members Bax and Bak induce the activation of caspase 9 through the release of cytochrome c from mitochondrial outer membrane permeabilization that then activates caspase 3 to induce cell death and caspase 7 to induce apoptotic cell detachment [41]. Cleaved caspase 7 levels were significantly lower (*p* = 0.003) and caspase 3 levels tended to be lower (*p* = 0.075) in the offspring of HFD fed dams, indicating a reduction in apoptosis in their mammary glands. Grape juice also significantly reduced caspase 7 (*p* = 0.013) and tended to reduce caspase 3 (*p* = 0.065) in the control offspring’s mammary glands but had no effect on apoptosis markers in the HFD offspring. Thus, maternal grape juice intake eliminated the difference in apoptosis markers between the control and HFD offspring (Figure 2A–C), but this was not due to grape juice-induced changes in the control group. Regardless of whether offspring were born to control or HFD fed dams, those also having been exposed to grape juice in utero exhibited lower levels of caspase 7 (0.546 ± 0.059) than offspring born to dams consuming tap water (0.746 ± 0.069), *p* = 0.045. We did not observe statistically significant differences among the groups in the levels of anti-apoptotic Bcl2, apoptotic Bax and Bcl2/Bax ratio (Figure 2A,D,E,F).

p21, p27 and p53. To further assess changes in cell proliferation and apoptosis in the mammary glands, we measured the levels of p21, p27 and p53. p21 is activated by caspase3 like kinases and p53 in association with induction of apoptosis, probably to assist in the execution of apoptosis by inhibiting cell cycle progression. p27 also controls cell cycle progression.

**Figure 3:**
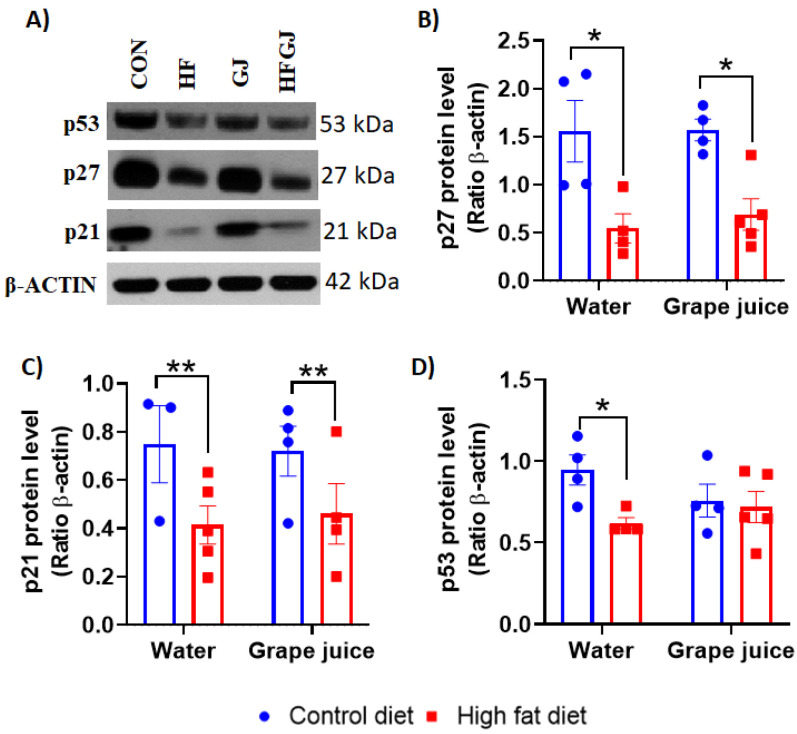
(**A**) Representative Western blots and quantitated data of (**B**) p27, (**C**) p21 and (**D**) p53 in mammary glands of 7-week-old female offspring from the control or HFD fed dams which either drank tap water or grape juice. The data are expressed as the mean ± SEM. * Statistically significant difference (*p* ≤ 0.05); ** *p* < 0.01 according to two-way ANOVA followed by Sidak′s test.

Protein levels of p21 (*p* < 0.05) and p27 (*p* < 0.05) were reduced in the HFD group’s offspring (Figure 3A–C), consistent with the increased mammary cell proliferation in these rats. Maternal grape juice had no effect on these endpoints. Protein levels of p53 were reduced in the HFD offspring (*p* = 0.045) (Figure 3A–C), in agreement with a lower level of apoptosis marker caspase 7. Grape juice had no effect on p53 levels in the HFD offspring, but it non-significantly reduced p53 levels in the control offspring, eliminating the difference between control and HFD offspring. These findings show that maternal HFD led to increased mammary cell proliferation, reduced cell cycle regulation and increased apoptosis in the offspring. Maternal grape juice also reduced apoptosis markers and p53 without affecting cell proliferation or cell cycle regulation in the control offspring. Thus, in the control offspring, grape juice may have reduced the levels of cellular stress.

**Figure 4:**
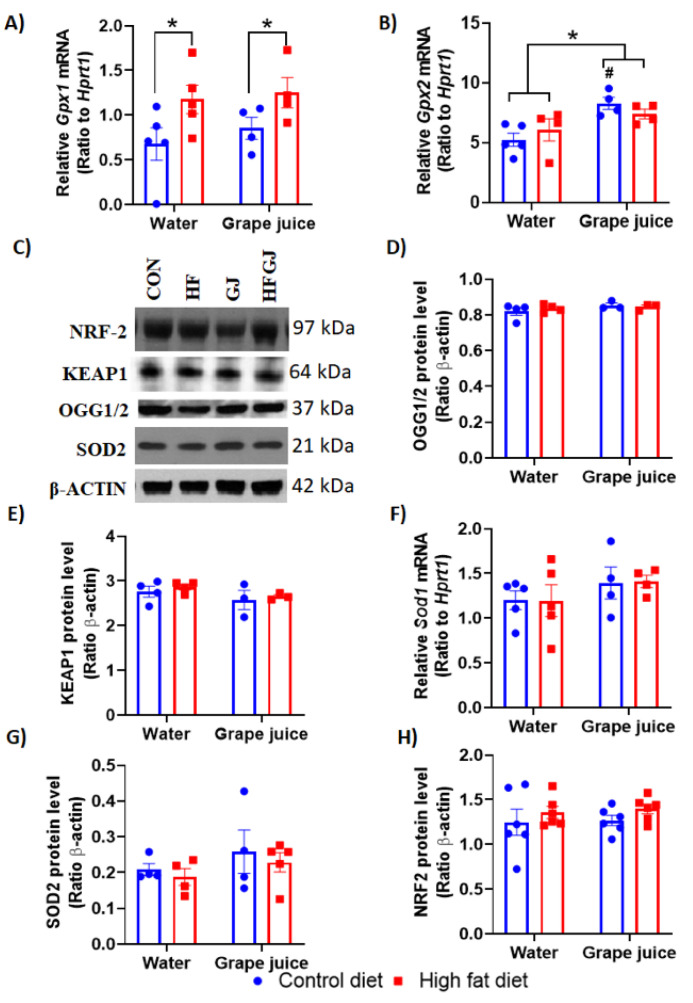
Quantitation of data from RT-qPCR analysis of (**A**) Gpx1, (**B**) Gpx2 and (**F**) Sod1 mRNA levels (**F**) and Western blot analysis of (**C**) OGG1/OGG2 (**D**), Keap1 (**E**), Sod2 (**G**) and Nrf-2 (**H**) protein levels in the mammary glands of 7-week-old female offspring from the control or HFD fed dams which either drank tap water or grape juice. * Statistically significant difference (*p* ≤ 0.05) according to two-way ANOVA followed by Sidak′s test. The data are expressed as the mean ± SEM. # *p* < 0.05, compared with the control offspring of dams drinking water.

Effects of maternal grape juice intake on oxidative stress markers in the offspring′s mammary gland. HFD is known to cause cellular oxidative stress [42], whilst grape juice reduces oxidative stress in multiple tissue and cell types [38,43,44]. We evaluated oxidative stress markers 8-oxoguanine DNA glycosylase 1 and 2 (OGG1/OGG2), nuclear factor erythroid 2–related factor 2 (NRF-2), superoxide dismutase 2, mitochondrial (SOD2) and Kelch-like ECH-associated protein 1 (KEAP1) (protein levels) and Sod1, glutathione peroxidase 1 (GPx1) and GPx2 (mRNA expression). Maternal HFD increased GPx1 expression (*p* = 0.028), but grape juice did not reduce it (Figure 4A). GPx2 expression was not different between the control and HFD offspring, but grape juice increased the expression of this gene in both groups (*p* = 0.018) (Figure 4B). Since GPx2 reduces oxidative stress-induced apoptosis [45], this finding is consistent with the effect of maternal grape juice on apoptosis marker caspase 7 in the control offspring. No other changes in the other oxidative stress markers were seen (Figure 4C–H).

### 3.2. Effects of Maternal Grape Juice Intake on Unfolded Protein Response in the Offspring′s Mammary Glands

**Figure 5:**
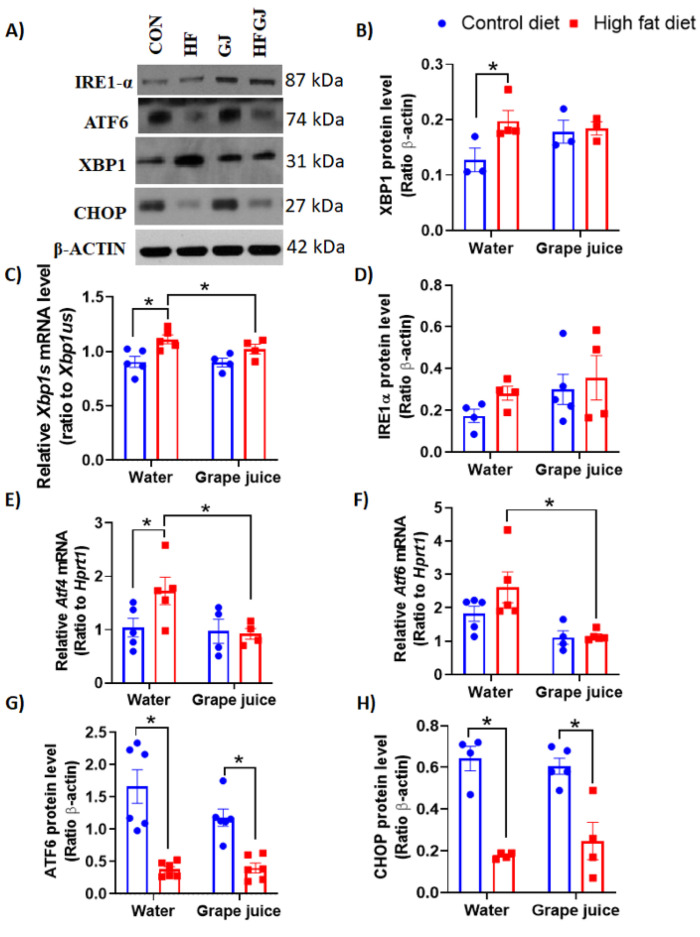
(**A**) Representative Western blots and quantitated data of (**B**) XBP1, (**D**) IR1-α, (**G**) ATF6 and (**H**) CHOP protein levels and data from RT-qPCR analysis of mRNA levels of (**C**) *Xbp1s* (ratio of spliced to unspliced version), (**E**) *Atf4* and (**F**) *Atf6* in the mammary glands of 7-week-old female offspring from the control or HFD fed dams which either drank tap water or grape juice. The data are expressed as the mean ± SEM. * Statistically significant difference (*p* ≤ 0.05) according to two-way ANOVA followed by Sidak′s test.

We observed that maternal HFD increased XBP1 protein levels (*p* = 0.05) (Figure 5A,B) and *Xbp1s* mRNA expression (*p* = 0.031) in the offspring’s mammary glands (Figure 5C). The increases were no longer seen if dams consumed grape juice during pregnancy. IRE1α or PERK protein levels were not significantly altered by maternal HFD or grape juice intake (Figure 5A,D). *Atf4* (*p* = 0.05) mRNA was elevated in the mammary glands of HFD offspring, and maternal consumption of grape juice reversed this increase (*p* = 0.020) (Figure 5E). Maternal grape juice intake also reduced the *Atf6* mRNA in HFD offspring (*p* = 0.05) (Figure 5F). CHOP and ATF6 protein levels, in contrast, were significantly reduced by in utero HFD exposure (*p* < 0.001 and *p* < 0.05), but grape juice did not modify the reduction (Figure 5A,G,H).

### 3.3. Effects of Maternal Grape Juice Intake on Erα, Her2, Pparγ and Irs1 Levels in the Offspring′s Mammary Glands

**Figure 6:**
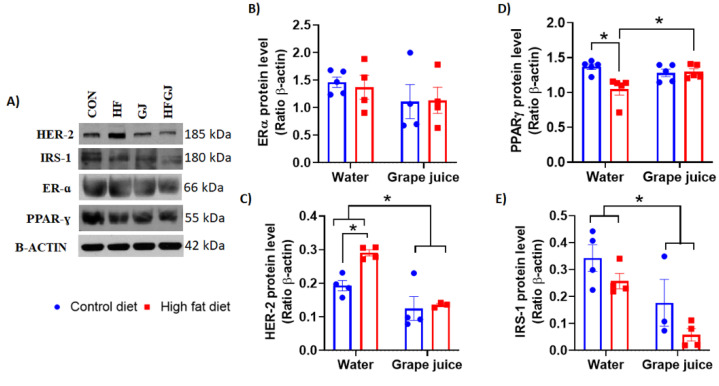
(**A**) Representative Western blots and quantitated data of (**B**) ERα, (**C**) HER2, (**D**) PPAR-Ɣ and (**E**) IRS-1 protein levels in the mammary glands of 7-week-old female offspring from the control or HFD fed dams which either drank tap water or grape juice. The data are expressed as the mean ± SEM. * Statistically significant difference (*p* ≤ 0.05). IRS-1 * *p* < 0.05 for water vs. grape juice.

We finally investigated whether maternal HFD and exposure to grape juice during pregnancy and lactation affected receptors in the offspring′s mammary glands that are commonly expressed in breast cancers, serve as treatment targets (ERα, HER2) and are linked to adipose differentiation and glucose homeostasis (PPARγ) and insulin and insulin-like growth factor signaling (IRS1). Neither maternal HFD nor grape juice affected ERɑ levels in the offspring′s mammary glands (Figure 6A,B). Two-way ANOVA indicated that Her2 protein levels were increased in the HFD offspring. This increase was reversed if the HFD dam also consumed grape juice (*p* < 0.01). Grape juice also reduced Her2 levels in the control offspring (*p* < 0.01) (Figure 6A,C).

PPARɣ levels were significantly downregulated in the HFD offspring (*p* < 0.05) (Figure 6A,D). Maternal grape juice intake reversed this downregulation in the HFD offspring (*p* < 0.05). IRS1 levels were not significantly different in the mammary glands of control and HFD offspring. Maternal grape juice exposure reduced IRS1 levels in both groups of offspring (*p* = 0.040) (Figure 6A,D).

## 4. Discussion

We investigated here whether maternal intake of grape juice during pregnancy can prevent some changes indicative of increased mammary cancer risk in the offspring of dams fed HFD during pregnancy and lactation. One such marker is the number of TEBs present in mammary glands at the time at which the gland is most susceptible to breast cancer initiation by carcinogens. Several previous studies have shown that the more TEBs that are seen in the mammary glands of 50-day-old rats, the higher their risk of developing mammary cancer upon exposure to, for example, 9,12-dimethylbenz[a]anthracene (DMBA) carcinogen [45,46]. TEBs contain a high number of mammary stem cells and proliferating epithelial cells, both of which are vulnerable to transformation into malignant cells [47]. As we have previously reported [14,15,48], maternal HFD that led to increased mammary cancer risk also increased TEBs in the offspring’s mammary glands. Consumption of grape juice by pregnant dams eliminated this difference in TEBs in the HFD offspring.

Other biomarkers of increased breast cancer risk that we assessed included epithelial cell proliferation and apoptosis markers. Ki-67 is a well-established marker of cell proliferation, and elevated levels in human breast cancers are predictive of poor prognosis [49,50]. We found that offspring of HFD fed dams exhibited increased levels of Ki-67 in their mammary glands. In addition, genes that control the cell cycle—p21 and p27—were downregulated in the HFD offspring. p21 and p27 are cyclin-dependent kinase inhibitors and key G1-checkpoint proteins which inhibit the cell cycle’s progression from the G1 to the S phase [50]. A reduction in the expression of these two genes is associated with more aggressive breast tumor phenotypes and metastasis [51]. The observed changes in Ki-67, p21 and p27 are in agreement with previous findings that maternal HFD increases mammary cancer risk among female offspring. Since we also found that maternal grape juice intake reversed the increase in Ki-67 levels in HFD offspring, although it did not upregulate p21 or p27, grape juice may protect offspring against the adverse effects of HFD during pregnancy on offspring’s increased mammary tumorigenesis.

Two markers of apoptosis—caspase 3 and 7—were expressed at lower levels in the mammary glands of HFD offspring than in the controls. This difference was no longer observed in the offspring of dams that also consumed grape juice. However, this result reflected a reduction of caspase 3 and 7 by grape juice in the control offspring, indicating that grape juice may have reduced apoptosis in the controls rather than promoting it in the HFD offspring. Similar data were obtained when p53 expression was assessed. p53 protein levels were significantly lower in the mammary glands of HFD offspring, but, due to maternal grape juice intake reducing p53 in the controls, this difference was no longer observed between control and HFD offspring of dams consuming grape juice. p53 has multiple roles: it is the guardian of the genome by protecting cells from DNA damage, and if damage takes place, p53 eliminates damaged cells by inducing apoptosis [52]. The reduction in apoptosis markers only in the control offspring of dams consuming grape juice may have been caused by the juice reducing the levels of oxidative stress [42] that in turn lowers the need for cell death. As apoptosis was already low in the HFD offspring, maternal grape juice intake did not further reduce it. We investigated here whether any antioxidant enzymes were differentially expressed in the mammary glands between control and HFD offspring. Neither Nrf2 nor Keap1 was affected by maternal obesity or grape juice intake, and antioxidant genes Ogg1, Ogg2, Sod1 or Sod2 were not affected either. Maternal HFD increased Gpx1 expression. Gpx1 is a ubiquitous cytosolic and mitochondrial antioxidant enzyme that converts intracellular H2O2 to water, using reduced glutathione as an electron donor. Its role in cancer remains unclear, as increased expression is linked to both reduced and increased susceptibility to cancer [53]. Another antioxidant enzyme, Gpx2, was not affected by maternal HFD. Maternal grape juice intake increased Gpx2 expression in the mammary gland, regardless of whether the dam consumed the control or HFD. Although elevated Gpx2 levels have been linked to increased breast cancer risk [54], Gpx2 may reduce the risk of breast cancer metastasis [55]. GPx2 also reduces oxidative stress-induced apoptosis [54], and the increase in GPx2 by maternal grape juice in the offspring may be linked to reduced apoptosis marker caspase 7 in the control offspring of grape juice-consuming dams. Taken together, maternal HFD and/or grape juice intake did not have major effects on antioxidant or ROS pathways, and it is unclear whether the few changes that were observed in the control or HFD offspring are linked to the potential effects of maternal grape juice on mammary cancer risk.

Obesity causes metabolic abnormalities which lead to EnR stress and chronic UPR [56,57,58]. EnR is a central organelle in eukaryotic cells: it stores and regulates calcium release, synthesizes lipids and folds proteins emerging from the ribosome. EnR stress activates UPR. We observed that maternal HFD increased the XBP1 protein levels and *Xbp1s* mRNA levels, and the levels of *Atf4* mRNA, but it reduced ATF6 and CHOP protein levels in the mammary gland. Since increased expression of CHOP may lead to cell cycle arrest and apoptosis [59], this finding is consistent with other markers of apoptosis also being inhibited (p53 and caspases 3 and 7) in the mammary glands of HFD offspring. Maternal grape juice intake reversed the increase in *Xbp1s* and *Atf4* mRNA levels in the HFD offspring but had no effect on reduced CHOP and ATF6 protein levels. Our data are in partial accordance with other studies showing that maternal HFD can lead to increased UPR in offspring [19,20].

To further understand how grape juice may protect offspring from maternal HFD, we assessed changes in obesity and breast cancer linked pathways that might intersect with grape juice’s biological effects. We investigated protein levels of ERα, Her2, PPARγ and IRS1. Only PPARγ was differentially expressed in the mammary glands between control and HFD offspring: significantly lower levels were observed in the mammary glands of HFD fed dams than in controls. PPARγ is a key regulator of adipocyte differentiation and glucose homeostasis [60]. PPARγ also regulates the expression of genes that control cell proliferation [61]. Previous studies have reported an increase in PPARγ expression by obesity in adipose tissue in an animal model [61] and in humans [62], perhaps reflecting an attempt of the increasing adiposity to maintain insulin sensitivity. No such increase was seen in the mammary tissue of offspring of HFD fed dams in our study. Instead, PPARγ was significantly reduced in the HFD offspring, and maternal grape juice intake reversed this reduction. The role of PPARγ expression in affecting breast cancer risk remains conflicting, but most studies suggest that low levels are associated with increased breast cancer risk [63,64]. If true, our finding that PPARγ expression was reduced in the HFD offspring and reversed by maternal grape juice intake suggests that PPARγ may protect against mammary cancer.

IRS1 was not affected by maternal HFD, but grape juice downregulated it in the mammary glands of control and HFD offspring. In a previous study, IRS1 was overexpressed in the mammary glands of the offspring of HFD dams [17]. IRS1 binds to insulin receptor and insulin-like growth factor receptor and, upon phosphorylation, activates their downstream signaling pathways, i.e., PI3K and ERK1/2. IRS1 can also be activated by cytokine receptors [65]. Furthermore, IRS1 can form a complex with ERα and regulate its transcription as well as interact with β-catenin to regulate epithelial mesenchymal transition and with cyclin D and c-myc, for example, to regulate cell proliferation [66]. It also has been shown that nuclear IRS1 attenuates DNA repair [67]. We observed that HER2 levels were increased in HFD offspring, and maternal grape juice downregulated the levels of this protein in both HFD and control offspring. Expression of this protein in breast cancer is linked to aggressive cancer cell progression, and its inhibition with monoclonal antibodies has revolutionized the treatment of HER2 amplified breast cancers. Similar to IRS1, its main downstream signaling pathways are PI3K and ERK1/2. The results of our study suggest that maternal intake of grape juice reduces the expression of IRS1 and HER2 in the normal mammary gland and thus is expected to inhibit mammary cell proliferation via the ERK1/2 pathway and resistance to apoptosis via the PI3K pathway.

In this study, we did not attempt to investigate whether there is a single compound in grape juice that might have been responsible for reversing the markers of increased mammary cancer risk in the HFD offspring. Two main reasons for this are the following: (1) during pregnancy and lactation, it is likely to be safer for a fetus to obtain nutrients from natural foods or drinks rather than from supplements; (2) individual ingredients in complex mixtures act differently than when they are taken in their natural food/drink matrix than when they are taken as a sole compound. Grape juice is an important source of different polyphenols, of which flavonoids [68], such as rutin and chlorogenic acid, were found to be the most abundant class in our study. Rutin has been reported to have antitumor effects in an in vivo model of breast cancer, acting as an antioxidant and inducing apoptosis [69]. Chlorogenic acid, also present in coffee, fruits and vegetables, has been associated with a lower incidence of breast cancer among postmenopausal women [70].

Our study has some potential weaknesses. Firstly, we were not able to investigate whether maternal grape juice intake reversed the increased mammary tumorigenesis in the HFD offspring. Secondly, the HFD fed pregnant dams failed to gain an excess amount of weight during pregnancy, although Wistar rats are sensitive to HFD-induced obesity. One possibility is that the HFD exposure was not long enough to result in significant weight gain. Furthermore, rats consumed a less energy-dense HFD than the control diet during pregnancy and consequently did not become obese. Since EnR stress in the HFD offspring might have, for example, led to NLRP3-inflammasome activation or a release of inflammatory mediators, and maternal obesity is known to increase inflammatory markers in the offspring [71,72], future studies should explore whether maternal grape juice might counteract increased inflammation in the HFD offspring.

In conclusion, our results indicate that maternal grape juice intake reversed the increase in TEBs and cell proliferation as well as the upregulation of human epidermal growth factor receptor 2 (HER2) and the downregulation of peroxisome proliferator-activated receptor γ (PPARγ) in the HFD offspring. It also reversed the elevation of UPR in HFD offspring′s mammary glands, i.e., prevented an increase in Xbp1 slicing and Atf4 and Atf6 mRNA levels. Thus, although we did not directly determine whether maternal grape juice intake can prevent increased mammary tumorigenesis in the offspring of dams fed HFD, its ability to reverse biomarkers of increased mammary cancer risk in these offspring—elevated number of TEBs, increased cell proliferation and alterations in UPR—suggest that it might. The possibility that the consumption of grape juice during pregnancy may reduce HFD offspring′s risk of developing breast cancer will be investigated in our future studies.

## Supplementary Materials

The following are available online at https://www.mdpi.com/2072-6643/12/8/2253/s1, Table S1: Average daily consumption of carbohydrates, protein and lipids in the pregnant and nursing dams fed control (CD) or HFD and tap water of grape juice (GJ), Table S2: Average daily intake of polyphenols from grape juice in the control diet or high fat diet (HFD) fed dams, Figure S1: (A) Offspring body weight (kg), evaluated after the lactation ended, (B) liver protein oxidation and (C) lipid peroxidation., Table S3: Antibodies used in Western blot experiments:, Table S4: Primers used for quantitative real-time PCR.
