# Supplementary material for: Effects of Maternal Grape Juice Intake on Unfolded Protein Response in the Mammary Glands of Offspring of High Fat Diet Fed Rat Dams
Source: Nutrients. 2020 Jul 28;12(8):2253. doi: 10.3390/nu12082253 (PMC7547380; doi:10.3390/nu12082253)
Supplement: Supplementary file 1 [file nutrients-12-02253-s001.pdf]

Table S1. Average daily consumption of carbohydrates, protein and lipids in the pregnant and nursing dams fed control (CD) or HFD and tap water of grape juice (GJ).

Average	CD (18.76±0.54)	HDF (11.51±0.63)*	CD+GJ (13.42±1.54)	HDF+GJ (9.09±1.54)*#
**Carbohydrates (g)**	12.38	2.87	8.85	2.27
**Protein (g)**	4.12	2.30	2.95	1.81
**Lipids (g)**	0.750	5.75	0.53	4.55

* $p<0.05$ compared with CD; #$p<0.05$ compared with all other groups. Data analyzed by 2-way ANOVA, followed by Sidak's post hoc test.

Table S2. Average daily intake of polyphenols from grape juice in the control diet or high fat diet (HFD) fed dams.

		**Control Diet**	**HFD**
	mg/L	Avg Consumption 23.42±2.61 mL/day	Avg Consumption 26.4±2.31 mL/day
**Total phenolic compounds**	2797	32.16*	36.61
**Total flavonoids content**	77.27	0.888	1.004
**Resveratrol**	0.508	0.0058	0.0066
**Epicatechin**	1.95	0.0224	0.0253
**Naringin**	3.37	0.0387	0.0438
**Rutin**	17.41	0.2002	0.2263
**Chlorogenic acid**	12.37	0.1422	0.1608

*Average daily consumption in mg.

Table S3. Antibodies used in Western blot experiments.

**Antibody**	**Company**	**Code**
Anti-Rabbit	Santa Cruz	sc-2030
Anti-Mouse	Santa Cruz	sc-2031
Anti-Goat	Santa Cruz	sc-2020
Actin	Santa Cruz	sc-1616
Cleaved Caspase 7	Cell Signaling	Cs-94915
Caspase 3	Santa Cruz	CPP324-1-18
Bcl-2	Enzo	83-8B
Bax	Upstar	06-499
p27	Santa Cruz	1641
p21	Oncogene	WAF-1/CIP1
p53	Cell Signaling	Cs-9282
Er-α	Vector	VPE613
Her-2	Invitrogen	MA5-13675
PPAR-Ɣ	Cell Signaling	CS-95425
IRS-1	Cell Signaling	D23G12
Xbp-1	Gen way Biotech	GWB-BACB31

Table S4. Primers used for quantitative real-time PCR.

Gene	Foward	Reverse
HPRT1	CCCCAAAATGGTTAAGGTTGC	AACAAAGTCTGGCCTGTATCC
GPX1	AACCTGACATAGAAACCCTGC	CAGTAATCACCAAGCCAATGC
GPX2	GTAGTTCTCGGCTTCCCTTG	GGTAAGACTAAAGGTGGGCTG
SOD1	TGTGTCCATTGAAGATCGTGTG	TCCCAGCATTTCCAGTCTTTG
ATF4	CCAAGCACTTCAAACCTCATG	GTCCATTTTCTCCAACATCCAATC
ATF6	GGAGAGGTGTCTGTTTCGGG	GTGGACTCCCAGTCTTCACC
IR1-α	TCATCACTTACCCCCTGAGCA	CTGGGTAAGGTCTCCGTGTG

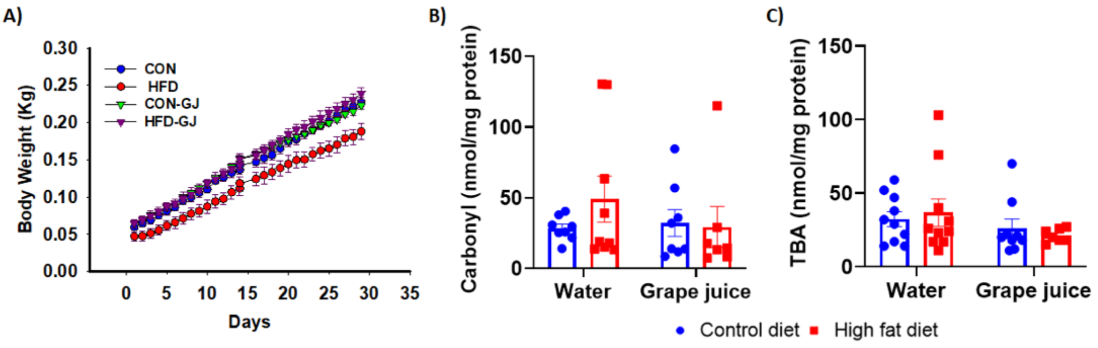

Figure S1. (A) Offspring's body weight (kg), evaluated after the lactation ended, (B) liver protein oxidation and (C) lipid peroxidation.
